# Association of caesarean section history with adverse pregnancy outcomes in placenta accreta spectrum: the mediating role of placenta previa

**DOI:** 10.7189/jogh.16.04045

**Published:** 2026-04-03

**Authors:** Yongdan Ma, Darui Gao, Ruilin Guo, Xin Wen, Huixia Yang, Jingmei Ma

**Affiliations:** Department of Obstetrics and Gynecology, Peking University First Hospital, Beijing, China

## Abstract

**Background:**

Placenta accreta spectrum (PAS) is a leading cause of severe maternal and neonatal complications, yet its risk pathways remain incompletely understood. We aimed to evaluate the association between the number of prior caesarean sections (CS) and adverse maternal and neonatal outcomes in PAS, and to quantify the mediating role of placenta previa (PP) in this relationship.

**Methods:**

Based on a retrospective cohort of 231 patients with PAS (61 with no prior CS, 124 with one CS, and 46 with ≥2 CS), we performed multivariate logistic regression to assess associations between CS history and pregnancy outcomes and conducted mediation analysis to estimate the proportion of the CS effect mediated by PP.

**Results:**

Patients with ≥2 prior CS had significantly higher rates of second-trimester surgical abortion, percreta, and higher PAS severity scores. While PP, embolisation, blood transfusion, and prolonged hospitalisation were more common in women with any CS history, their frequencies did not increase proportionally with the number of CS. Multivariate analysis demonstrated that a higher number of prior CS was independently associated with increased risks of invasive PAS, massive intraoperative haemorrhage and transfusion, and preterm delivery. Mediation analysis showed that PP contributed to 8.62% of the association between CS history and invasive PAS, 16.93% for transfusion, 32.83% for preterm birth, and 11.40% for neonatal intensive care unit admission.

**Conclusions:**

A higher number of previous CS procedures in our sample was associated with higher risks of several adverse maternal outcomes in PAS. Neonatal outcomes, however, were not significantly influenced by the number of prior CS procedures and were more strongly affected by the gestational age at delivery. These relationships were partially mediated by PP, highlighting its important, but incomplete role in the pathway linking prior CS to PAS-related complications.

Placenta accreta spectrum (PAS) disorders, including accreta, increta, and percreta, are characterised by abnormal trophoblast invasion into the myometrium [1,2]. Despite their relatively low incidence [3], PAS conditions remain a major cause of severe postpartum haemorrhage, hysterectomy, and maternal morbidity and mortality [1,4]. Accurate prenatal diagnosis is essential for preventing such cases, but challenging; the sensitivity of ultrasound remains only 50–60% [5–9]. Consequently, approximately half of PAS cases remain undiagnosed before delivery [5,10], limiting timely preparation and multidisciplinary management.

Caesarean section (CS) is the most well-established risk factor for PAS, although other intrauterine procedures that injure the endometrium or myometrium, such as myomectomy [11], hysteroscopic adhesiolysis [12], and curettage, also increase PAS risk [13,14]. However, how pregnancy characteristics and clinical outcomes vary across PAS populations with different intrauterine procedures histories remains insufficiently defined.

Importantly, previous CS markedly increases the likelihood of placenta previa (PP) [15], and both CS and PP independently elevate the risk of PAS [10,16]. Yet, limited evidence exists on how PP contributes to adverse maternal and neonatal outcomes specifically among PAS patients with a history of CS, and on whether it mediates the association between prior CS and adverse outcomes. This knowledge gap restricts early identification of high-risk PAS cases and limits preoperative planning.

To address this gap, we conducted a retrospective study of PAS patients to examine how increasing numbers of prior CS relate to maternal and neonatal outcomes and to evaluate the mediating role of PP in the pathway from previous CS to adverse clinical outcomes. Our findings could support improved risk stratification and resource allocation in the management of PAS.

## METHODS

We conducted this study at Peking University First Hospital, whose obstetrics department is one of the region’s referral centers for the management of high-risk pregnancies and delivers more than 6000 babies each year. We report our findings in accordance with the STROBE guidelines (File S1 in the **Online Supplementary Document****)**.

### Diagnosis and classification of PAS

All patients included in this study had a confirmed PAS based on either intraoperative clinical assessment, histopathological examination, or both. The classification of PAS was according to the FIGO 2019 consensus [17], as follows:

- FIGO grade 1 (placenta accreta): villous tissue adherent to the myometrium without invasion.
- FIGO grade 2 (placenta increta): placental invasion into the myometrium without breaching the serosa.
- FIGO grade 3 (placenta percreta): invasion through the serosa, with or without involvement of adjacent organs.

Both the increta and the percreta are considered invasive types [17].

### Inclusion and exclusion criteria

We included patients with a confirmed diagnosis of PAS (clinical, pathological, or both), clear FIGO grading available based on surgical documentation, and complete obstetric and surgical records. We excluded patients with multiple gestation, missing or inconsistent key variables, incomplete intraoperative or medical documentation that prevented reliable PAS confirmation, and, for neonatal outcome analyses, pregnancies ending in curettage or termination.

### Data collection

Trained medical professionals reviewed all electronic medical records and extracted clinical information using a standardised data abstraction form. Variables collected included:

- demographic characteristics;
- reproductive and uterine surgical history: gravidity, parity, abortion, previous CS, curettage, hysteroscopic surgery, myomectomy, and intrauterine adhesions;
- characteristics of current pregnancy: gestational age, mode of conception, prenatal haemorrhage, placenta previa, PAS type, PAS score based on previously published scoring systems [18,19] (Table S1 in the **Online Supplementary Document**);
- perioperative events: intraoperative blood loss, transfusion, uterine artery embolisation or balloon intervention, adjacent organ damage, disseminated intravascular coagulation, haemorrhagic shock, hysterectomy, postpartum haemorrhage, and length of hospital stay;
- neonatal outcomes: gestational age at delivery, birth weight, neonatal asphyxia, Apgar score 1/5/10 minutes, and neonatal intensive care unit (NICU) admission.

Cases with missing values in key variables or incomplete surgical or medical documentation were excluded a priori to minimise misclassification. Data abstraction was performed by trained reviewers and independently verified by a second reviewer to reduce misclassification and bias. Data completeness was verified before analysis.

### Data analysis

After testing for normality using the Shapiro-Wilk test, we summarised continuous variables as means and standard deviations if normally distributed and as medians and interquartile ranges otherwise. Likewise, we compared groups using ANOVA for normally distributed variables and the Kruskal-Wallis test for non-normally distributed variables. When an overall group difference was detected, we performed *post-hoc *pairwise comparisons using the Bonferroni correction. We presented categorical variables frequencies and percentages and compared them using the χ^2^ or Fisher’s exact test, as appropriate.

We constructed multivariate logistic regression models to evaluate the association between the number of previous CS and adverse maternal or neonatal outcomes, reporting the results as adjusted odds ratios (aORs) with 95% confidence intervals (CIs). Model 1 was adjusted for sociodemographic characteristics and established PAS risk factors identified in prior studies, including maternal age ≥35 years, history of induced abortion, history of curettage, history of intrauterine adhesion treatment, history of hysteroscopic surgery, history of myomectomy, placenta previa, current assisted reproductive technology, and gestational age at delivery [19–21]. Model 2 was adjusted for maternal factors associated with neonatal outcomes reported in previous literature, including maternal age ≥35 years, placenta previa, current assisted reproductive technology, and gestational age at delivery [22–24]. We assessed all models for multicollinearity using the variance inflation factor test (Tables S2 and S3 in the **Online Supplementary Document**).

We performed causal mediation analysis to examine the mediating role of placenta previa in the association between presence or absence of prior CS and adverse outcomes, given that CS history occurred prior to the index pregnancy, PP was diagnosed during the current pregnancy, and adverse outcomes occurred at delivery, the temporal ordering necessary for mediation (exposure → mediator → outcome) was inherently satisfied in this cohort design. We conducted a quasi-Bayesian Monte Carlo method with 1000 simulations according to normal approximation to get a stable result [25,26]. Here, the average causal mediation effect (ACME) reflects the indirect effect of placenta previa (mediator) on the relationship between CS history (exposure) and PAS maternal adverse outcome (outcome). Meanwhile, the average direct effect (ADE) indicates the direct effect of exposure and outcome. To evaluate the mediated proportion attributable to placenta previa, we employed the calculation of ACME relative to the total effect (ACME + ADE) [27]. We assessed key assumptions required for causal mediation, including correct model specification and absence of mediator-outcome confounding after adjustment for covariates used in the multivariable regression models. Wider CIs around mediation proportions were interpreted as indicating a high degree of statistical uncertainty.

We conducted all analyses using Prism, version 9.0 (GraphPad Software, LLC, USA), SAS, version 9.4 (SAS Institute Inc, Cary, USA) and *R*, version 3.4.0 (R Foundation, Vienna, Austria), with the *R* ‘mediation’ package applied for the mediation analysis. A two-tailed *P* value <0.05 was considered statistically significant.

## RESULTS

### Demographic and clinical characteristics

**Figure 1:**
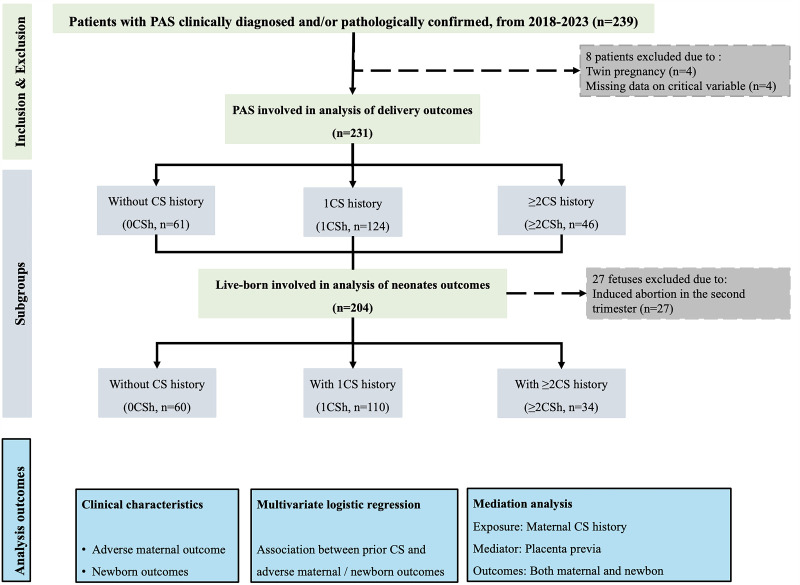
Flow diagram of patients included in this study cohort.

We included 231 patients with PAS (**Figure 1****,** **Table 1**), of whom 61 had prior CS (0 CSh), 124 had one prior CS (1 CSh), and 46 had two or more CS (≥2 CSh). Maternal age and the prevalence of advanced maternal age and prior abortions were comparable across groups. In contrast, patients in the 0 CSh group had higher frequencies of prior uterine procedures such as intrauterine adhesions treatment (n/N = 8/61, 13.1%), hysteroscopic surgeries (n/N = 18/61, 29.5%), and myomectomy (n/N = 6/61, 9.8%).

**Table 1:** Clinical characteristics of the study population stratified by history of prior CS (n = 231)*

Variables	0CSh (n = 61)	1CSh (n = 124)	≥2CSh (n = 46)	*P-*value
Age, x̄ (SD)	34.25 (4.45)	34.63 (4.15)	33.93 (4.69)	0.623
Age ≥35 y	30 (49.2)	69 (55.6)	18 (39.1)	0.155
Gravidity ≥5	19 (31.1)	6 (4.8)	0 (0.0)	<0.001
Parity ≥5	1 (1.6)	4 (3.2)	46 (100.0)†	<0.001
Abortion ≥5	16 (26.2)	53 (42.7)	21 (45.7)	0.056
Induced abortion ≥5	13 (21.3)	45 (36.3)	14 (30.4)	0.117
Curettage ≥1	9 (14.8)	17 (13.7)	5 (10.9)	0.835
IUA≥1	8 (13.1)	2 (1.6)‡	0 (0.0)‡	<0.001
Hysteroscopy ≥1	18 (29.5)	8 (6.5)‡	4 (8.7)‡	<0.001
Myomectomy ≥1	6 (9.8)	4 (3.2)‡	0 (0.0)‡	0.032

SD – standard deviation, IUA – intrauterine adhesion, x̄ – mean

*Presented as n (%) unless specified otherwise.

†*P* < 0.05 *vs*. 0CSh and *vs*. 1CSh.

‡*P* < 0.05 *vs*. 0CSh.

### Maternal outcomes and postoperative characteristics

Use of assisted reproduction was common in the 0 CSh group, and most patients underwent caesarean delivery in the third trimester (98.4%). The distribution of PAS subtypes differed by CS history: invasive forms were substantially more common in the ≥2 CSh group (93.5%), accompanied by higher PAS scores (Table S4 in the **Online Supplementary Document**).

Women with any history of CS demonstrated greater perioperative complexity, including higher requirements for abdominal aortic balloon occlusion, blood transfusion, and prolonged hospitalisation. Postpartum haemorrhage occurred more frequently in the 1 CSh group (59.7%), while patients with ≥2 CSh had the highest utilisation of uterine artery embolisation (10.9%).

### Association between prior CS and adverse maternal outcomes

Adjusted analyses showed that prior CS was associated with an increased likelihood of invasive PAS and haemorrhagic complications (**Table 2**). Compared with the 0 CSh group, patients with 1 CSh had higher odds of invasive PAS (aOR = 7.33; 95% CI = 3.55–15.60), while the ≥2 CSh group showed an even stronger association (aOR = 18.41; 95% CI = 7.50–46.93). The risk of PP increased with CS history for both the 1 CSh (aOR = 2.67; 95% CI = 1.23–5.79) and ≥2 CSh groups (aOR = 3.11; 95% CI = 1.12–8.66). The risk of intraoperative blood loss >1500 mL followed a similar pattern for the 1 CSh (aOR = 3.14; 95% CI = 1.06–9.25) and ≥2 CSh groups (aOR = 3.52; 95% CI = 1.06–11.69), paralleling higher transfusion requirements. Following further adjustment for gestational age at delivery, the findings showed that previous CS significantly increased the risk of adverse maternal outcome (Table S5 in the **Online Supplementary Document**).

**Table 2:** Association between prior CS and adverse maternal outcomes*

Variables	0CSh (n = 61)	1CSh (n = 124)		≥2CSh (n = 46)	
		**OR (95%CI)**	**aOR (95%CI)**	**OR (95%CI)**	**aOR (95%CI)**
Invasive PAS (increta/percreta)	ref	7.87 (4.18–15.28)	7.33 (3.55–15.60)	20.01 (8.91–46.72)	18.41(7.50–46.93)
Placenta previa	ref	3.26 (1.68–6.29)	2.67 (1.23–5.79)	4.30 (1.73–10.73)	3.11 (1.12–8.66)
Blood loss volume >1500 mL	ref	3.60 (1.42–9.13)	3.14 (1.06–9.25)	4.01 (1.4–11.47)	3.52 (1.06,11.69)
Blood transfusion	ref	3.34 (1.76–6.36)	3.06 (1.46–6.42)	4.84 (2.11–11.1)	4.10 (1.63,10.34)
Postpartum haemorrhage	ref	2.28 (1.22–4.27)	2.21 (1.07–4.60)	1.68 (0.78–3.65)	1.73 (0.72–4.17)

CI – confidence interval, OR – odds ratio, ref – reference

*Adjusted for maternal age ≥35 y, history of induced abortion, history of curettage, history of IUA treatment, history of hysteroscopic surgery, history of myomectomy, placenta accreta (when analysis the aOR of other risk factor), and current assisted reproductive technology.

### Neonatal outcomes

Among 204 live-born singleton infants (Table S6 in the **Online Supplementary Document**), the incidence of neonatal asphyxia and Apgar scores at 10 minutes were comparable across groups. However, prematurity and NICU admission were more common in infants born to mothers with prior CS. After adjusting for confounders analyses (**Table 3**) indicated a progressive increase in preterm delivery with additional CS exposure fort the 1 CSh (aOR = 2.60; 95% CI = 1.13–5.96) and ≥ 2CSh groups (aOR = 5.96; 95% CI = 1.24–28.75), with similar patterns for low birth weight (1 CSh: aOR = 2.40; 95% CI = 1.11–5.16, ≥ 2CSh: aOR = 3.92; 95% CI = 1.50–10.25, respectively). NICU admission was also more frequent for the 1 CSh (aOR = 5.94; 95% CI = 2.80–12.62) and ≥2 CSh groups (aOR = 7.28; 95% CI = 2.49–21.31).

**Table 3:** Association between prior CS and adverse outcomes among live-born infants*

Variables	0CSh (n = 60)		1CSh (n = 110)			≥2CSh (n = 34)	
	**OR (95%CI)**		**aOR (95%CI)**	**OR (95%CI)**		**aOR (95%CI)**
Preterm delivery	ref	3.65 (1.74–7.68)		2.6 (1.13–5.96)	9.95 (2.17–45.49)		5.96 (1.24–28.75)
Low birth weight	ref	2.16 (1.07–4.33)		2.4 (1.11–5.16)	3.80 (1.55–9.29)		3.92 (1.50–10.25)
Asphyxia	ref	2.59 (1.05–6.34)		2.32 (0.89,6.02)	1.96 (0.62–6.17)		1.61 (0.49–5.31)
NICU administration	ref	6.31 (3.16,12.63)		5.94 (2.80,12.62)	8.67 (3.10–24.25)		7.28 (2.49–21.31)

CI – confidence interval, NICU – neonatal intensive care unit, OR – odds ratio, ref – reference

*Adjusted for maternal age ≥35 y, placenta previa, and current assisted reproductive technology.

In contrast, after adjusting for confounders including gestational age (Table S7 in the **Online Supplementary Document**), NICU admission remained significantly associated with maternal CS history for the 1 CSh (aOR = 3.42; 95% CI = 1.39–8.42), while low birth weight and neonatal asphyxia showed no significant association.

### Mediation effect of placenta previa

**Figure 2:**
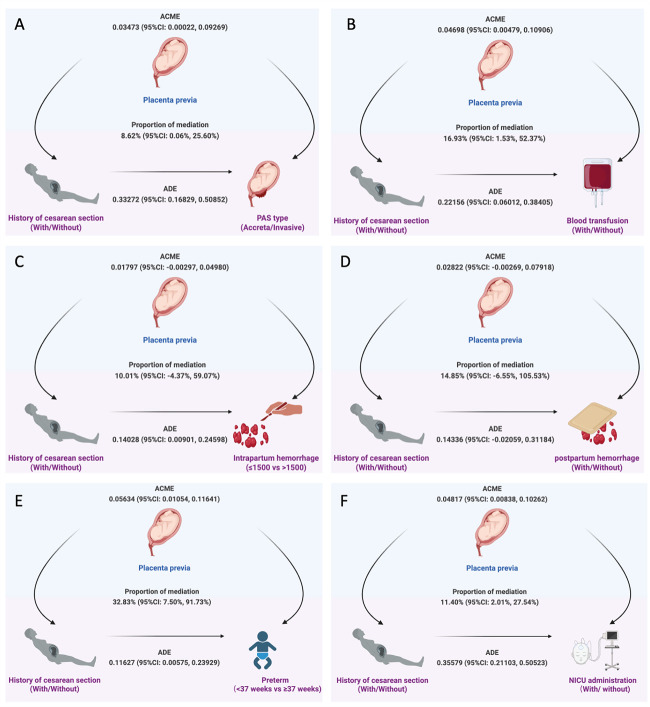
The mediation effects of placenta previa on the association between CS history and adverse pregnancy outcomes in PAS. **Panel A**. PAS type. **Panel B**. Blood transfusion. **Panel C**. Intrapartum hemorrhage. **Panel D**. Postpartum hemorrhage. **Panel E**. Preterm delivery. **Panel F**. NICU administration. ACME – average causal mediation effect, ADE – average direct effect, PAS – placenta accreta spectrum, NICU – neonatal intensive care unit.

Given that PP is both a known risk factor for and a frequent adverse outcome of PAS, we further explored the mediating role of PP in the association between prior CS and adverse pregnancy outcomes (**Figure 2**). We found that PP partially explained the association between prior CS and selected maternal and neonatal outcomes. PP mediated 8.62% (95% CI = 0.06–25.6) of the effect of CS history on invasive PAS and 16.93% (95% CI = 1.53–52.37) of the effect on blood transfusion. For neonatal outcomes, PP mediated 32.83% (95% CI = 7.50–91.73) of the association between CS history and preterm birth, and 11.40% (95% CI = 2.01–27.54) of the association with NICU admission. We observed no significant mediation for intraoperative blood loss >1500 mL or postpartum haemorrhage.

## DISCUSSION

In this retrospective cohort of patients with PAS, we found that increasing numbers of prior CS were associated with higher risks of invasive PAS, PP, massive intraoperative haemorrhage, transfusion, and preterm delivery. Through mediation analysis, we further demonstrated that PP emerged as a significant mediator linking CS history to both maternal and neonatal outcomes. These findings support the hypothesis that PP contributes to the pathway through which previous CS is associated with PAS severity and adverse clinical outcomes.

Among patients without a history of CS, accreta was the predominant subtype, and maternal morbidity was comparatively lower. In contrast, those with two or more prior CS showed higher rates of invasive PAS and markers of increased clinical complexity, consistent with existing literature [28]. These associations should, however, be interpreted cautiously due to the retrospective design, which precludes establishing causality and may be influenced by residual confounding or selection bias.

The timing of delivery in patients with PAS remains a critical consideration for obstetric management. Previous studies have identified PAS as an independent risk factor for late preterm birth and perinatal mortality [29]. It has been recommended that patients with suspected PAS deliver at a tertiary care centre between 34 and 36 weeks of gestation [30]. In our cohort, prior caesarean delivery was significantly associated with lower birth weight and increased NICU admission. However, after further adjusting for gestational age, we found no significant association between prior caesarean delivery and neonatal outcomes among PAS pregnancies, indicating that these relationships were largely driven by differences in gestational age at delivery. These findings suggest that, even among PAS patients, prior caesarean history and PAS disease characteristics may have limited direct impact on neonatal outcomes when delivery timing is accounted for. Rather, neonatal outcomes appear to be predominantly influenced by gestational age at birth. This observation is consistent with recent evidence showing that, in the absence of additional maternal or foetal indications for early delivery, postponing delivery may improve neonatal outcomes [31]. Collectively, these results underscore the importance of managing PAS cases within high-level care facilities capable of providing coordinated maternal and neonatal support to optimise both safety and outcomes [32].

The mediation analysis indicated that PP partially mediated the associations between CS history and several maternal and neonatal outcomes, including invasive PAS, transfusion, preterm birth, and NICU admission. Notably, PP did not significantly mediate the relationship between CS history and intrapartum blood loss >1500 mL or postpartum haemorrhage, indicating that other factors, such as surgical expertise, uterine contractility, and delivery timing, likely play substantial roles. However, these mediation effects should be interpreted cautiously, some mediation estimates exhibited wide confidence intervals, reflecting sample size limitations and residual confounding. Thus, the mediation findings are best viewed as exploratory, offering a potential conceptual framework for future prospective studies rather than definitive causal pathways.

These findings have direct clinical relevance. Because PP amplifies risk in patients with prior CS, targeted antenatal surveillance and risk stratification may help identify high-risk PAS cases earlier. Delivery in tertiary centres with multidisciplinary teams remains essential to optimising outcomes. Furthermore, our results reinforce the importance of minimising non-medically indicated CS to reduce cumulative risk in future pregnancies.

### Strengths and limitations

A major strength of this study is its focus on patients with PAS stratified by the number of previous CS, which enables a more detailed assessment of potential dose-response relationships between surgical history and maternal and neonatal outcomes. The inclusion of a wide range of clinically relevant outcomes provides a comprehensive evaluation of both maternal morbidity and neonatal health. Furthermore, the mediation analysis quantifying the contribution of PP offers additional insight into the pathways linking CS history with PAS severity and perinatal outcomes, supporting more refined clinical risk stratification.

Several limitations should also be acknowledged. First, this single-centre study was conducted at a tertiary referral hospital, which may introduce referral bias and limit generalisability, as patients with more severe or complex conditions are more likely to be referred. Second, compared with other obstetric conditions, PAS is relatively rare [3,33], which resulted in modest subgroup sample sizes, particularly among women with three or more prior CS, and consequently prevented us from performing more granular stratified analyses. Third, although we adjusted for key confounders, including gestational age for neonatal outcomes, the limited sample size in the ≥2CSh group led to wide CIs and reduced the statistical stability of both multivariable estimates and mediation analyses. Larger, prospective studies are needed to verify these associations. Fourth, although mediation analysis was conducted to explore the potential role of PP in the association between prior CS and adverse outcomes, the wide CIs of several mediation estimates reflect limited statistical power and potential residual confounding. Because the available sample size was insufficient to support a reliable sensitivity analysis for unmeasured mediator-outcome confounding, the mediation findings should be interpreted as exploratory and primarily hypothesis-generating. Fifth, although our multivariable models accounted for established risk factors, residual confounding from variables that were unmeasured or only imperfectly captured cannot be excluded. Factors such as the surgical technique used in previous caesarean deliveries, the depth of prior curettage, interpregnancy interval, and other clinical decision-related variables may still influence the associations observed. Sixth, our analysis focused on the number of previous CS rather than the indications for those procedures, which may also represent an important source of bias. For example, women with PP or other high-risk conditions are more likely to undergo repeat CS, and a history of PP increases the risk of PP in subsequent pregnancies, which may further affect maternal and neonatal outcomes in the current pregnancy.

## CONCLUSIONS

Increasing numbers of prior CS in our sample were associated with greater PAS severity and higher risks of adverse maternal and neonatal outcomes in this cohort. PP appeared to partially mediate these associations, although the mediation findings were exploratory and require validation in larger prospective studies. These results highlight the importance of comprehensive antenatal evaluation, timely referral to specialised centres, and the avoidance of non-medically indicated primary CS whenever possible. Future multicentre studies with more robust methodological frameworks are needed to further elucidate the pathways linking CS history, PP, and PAS-related morbidity.

## Additional material


Online Supplementary Document
