# Supplementary material for: Association of caesarean section history with adverse pregnancy outcomes in placenta accreta spectrum: the mediating role of placenta previa
Source: J Glob Health. 2026 Apr 3;16:04045. doi: 10.7189/jogh.16.04045 (PMC13045927; doi:10.7189/jogh.16.04045)
Supplement: Online Supplementary Document [file jogh-16-04045-s001.pdf]

Supplement to: Ma Y, Gao D, Guo R, Wen X, Yang H, Ma J. Association of caesarean section history with adverse pregnancy outcomes in placenta accreta spectrum: the mediating role of placenta previa. J Glob Health. 2026;16:04045.

Table S1. Placenta accreta scoring system.
Table S2. Variance inflation factor (VIF) analysis for multivariable models in Table 3.
Table S3. Variance inflation factor (VIF) analysis for multivariable models in Table 4.
Table S4. Clinical characteristics and perioperative outcomes among patients with PAS (N=231).
Table S5. Association between prior CS and adverse maternal outcomes (adjusted for gestational age).
Table S6. Neonatal clinical characteristics among PAS patients with live births (N = 204).
Table S7. Association between maternal cesarean section history and adverse neonatal outcomes (adjusted for gestational age).
Checklist S1. STROBE reporting checklist

Table S1. Placenta accreta scoring system

	Items	0 point	1 point	2 points	3 points
**Risk factors**	Age	<35	≥35	—	—
	Gravidity	No	Yes	—	—
	Previous abortion or miscarriage	No	Yes	—	—
	Previous cesarean sections	0	1	2	≥3
	Placenta previa	No	Marginal	Partial	Complete
**Ultrasound features**	Multiple vascular lacunae	No	Yes	Swiss-Cheese appearance	—
	Disruption of myometrium	Continuous	Disrupted	Disappeared	—
	Boundaries between the placenta and the myometrium	Clear	Obscure	Disappeared	—
	Uterine serosa-posterior bladder wall interface	Clear	Obscure	Disappeared	—
	Sub-placental hypervascularity	No	Turbulent flow	Bridging vessels	—
	Extending into the bladder	No	Yes	—	—
	Extending into the cervix	No	Yes	—	—

Table S2. Variance inflation factor (VIF) analysis for multivariable models in Table 3

	Variance Inflation Factor (VIF)				
	**Invasive PAS (Increta/ Percreta) as outcome**	**Placenta previa as outcome**	**Blood loss volume >1500 mL as outcome**	**Blood transfusion as outcome**	**Postpartum Hemorrhage as outcome**
**Prior C-Sections**	1.074	1.083	1.072	1.073	1.083
**Age ≥35**	1.071	1.076	1.040	1.059	1.060
**Induced abortion**	1.038	1.061	1.025	1.047	1.037
**Curettage**	1.088	1.086	1.039	1.074	1.069
**IUA**	1.336	1.339	1.400	1.318	1.311
**Hysteroscopy**	1.287	1.319	1.366	1.285	1.284
**Myomectomy**	1.034	1.041	1.047	1.053	1.071
**Current ART**	1.100	1.130	1.000	1.081	1.113

Table S3. Variance inflation factor (VIF) analysis for multivariable models in Table 4

	Variance Inflation Factor (VIF)			
	**Preterm delivery as outcome**	**Low birth weight as outcome**	**Asphyxia as outcome**	**NICU administration as outcome**
**Prior C-Sections**	1.042	1.055	1.034	1.043
**Age ≥35**	1.041	1.035	1.028	1.044
**Current ART**	1.098	1.123	1.068	1.084

Table S4. Clinical characteristics and perioperative outcomes among patients with PAS (N=231)

Variables	0CSh (N=61)	1CSh (N=124)	≥2CSh (N=46)	*P*-Value
**Current pregnancy**				
IVF (N, %)	19 (31.1)	6 (4.8) *	0 (0.0) *	<0.001
Prenatal hemorrhage (N, %)	20 (32.8)	34 (27.4)	6 (13.0)	0.061
Curettage (N, %)	1 (1.6)	2 (1.6)	1 (2.2)	
Second trimester surgical abortion (N, %)	0 (0.0)	12 (9.7) *	11 (23.9) *,†	0.002
Cesarean section (N, %)	60 (98.4)	110 (88.7)	34 (73.9) *	
**PAS type**				
Accreta (N, %)	35 (57.4)	21 (16.9) *	3 (6.5) *	
Increta (N, %)	24 (39.3)	57 (46.0)	16 (34.8)	<0.001
Percreta (N, %)	2 (3.3)	46 (37.1) *	27 (58.7) *,†	
**Placenta previa (N, %)**	32 (52.5)	97 (78.2) *	38 (82.6) *	<0.001
**PAS Score [Media, (P25, P75)]**	8.00 [5.00, 10.00]	11.00 [8.00, 14.00] *	13.00 [11.00, 16.00] *,†	<0.001
**Peri-operational events**				
**AAE (N, %)**	6 (9.8)	40 (32.3) *	14 (30.4) *	0.004
**UAE (N, %)**	0 (0.0)	4 (3.2)	5 (10.9) *	0.014
**UAL (N, %)**	40 (65.6)	84 (67.7)	30 (65.2)	0.932

**Blood loss volume [ml, Media, (P25, P75)]**	700.00 [500.00, 1200.00]	1000.00 [600.00, 1600.00] *	1000.00 [600.00, 1800.00]	0.003
**Blood transfusion (N, %)**	21 (34.4)	79 (63.7) *	33 (71.7) *	<0.001
**Hysterectomy (total or partial) (N, %)**	0 (0.0)	12 (9.7) *	4 (8.7)	0.045
**Complications**				
**DIC (N, %)**	0 (0.0)	4 (3.2)	1 (2.2)	0.366
**ATR (N, %)**	0 (0.0)	1 (0.8)	0 (0.0)	0.648
**Shock (N, %)**	0 (0.0)	4 (3.2)	2 (4.3)	0.305
**Bladder injury (N, %)**	0 (0.0)	4 (3.2)	4 (8.7)	0.05
**Ureteral injury (N, %)**	61 (100.0)	124 (100.0)	46 (100.0)	NA
**Intestinal injury (N, %)**	0 (0.0)	2 (1.6)	0 (0.0)	0.419
**Postpartum Hemorrhage (N, %)**	24 (39.3)	74 (59.7) *	24 (52.2)	0.033
**Length of stay [days, Media, (P25, P75)]**	7.00 [5.00, 10.00]	9.00 [7.00, 14.00] *	10.50 [7.25, 15.00] *	<0.001

IVF – in-vitro fertilization, AAE – Abdominal Aortic Embolism, UAE – Uterine Artery Embolism, UAL – Uterine artery ligation, DIC – Disseminated Intravascular Coagulation, ATR – Acute transfusion reaction, * P <0.05 vs 0CSh; † P <0.05 vs 1CSh.

Table S5. Association between prior CS and adverse maternal outcomes (adjusted for gestational age)

	0CSh N=61	1CSh N=124		≥2CSh N=46	
		OR (95%CI)	aOR (95%CI)	OR (95%CI)	aOR (95%CI)
**Invasive PAS (Increta/ Percreta)**	ref	7.87 (4.18, 15.28)	**6.55 (3.15, 14.03)**	20.01 (8.91, 46.72)	**14.71 (5.85, 38.25)**
**Placenta previa**	ref	3.26 (1.68, 6.29)	**2.27 (1.02, 5.07)**	4.30 (1.73, 10.73)	2.33 (0.80, 6.80)
**Blood loss volume >1500 mL**	ref	3.60 (1.42, 9.13)	**3.38 (1.12, 10.18)**	4.01 (1.4, 11.47)	**4.05 (1.16, 14.17)**
**Blood transfusion**	ref	3.34 (1.76, 6.36)	**3.37 (1.58, 7.20)**	4.84 (2.11, 11.1)	**5.05 (1.88, 13.54)**
**Postpartum Hemorrhage**	ref	2.28 (1.22, 4.27)	**2.75 (1.29, 5.88)**	1.68 (0.78, 3.65)	**2.60 (1.01, 6.70)**

OR, odds ratio; CI, confidence interval; adjusted for maternal age ≥35 years, history of induced abortion, history of curettage, history of IUA treatment, history of hysteroscopic surgery, history of myomectomy, PP (when analysis the aOR of other risk factor), current assisted reproductive technology (ART), and gestational age at delivery.

Table S6. Neonatal clinical characteristics among PAS patients with live births (N = 204)

	0 CSh<br>N=60	1CSh<br>N=110	≥2CSh<br>N=34	P value
**Delivery weeks**				
28-<34 (N, %)	6 (10.0)	24 (21.8)	9 (26.5)	**<0.001**
34-<37 (N, %)	31 (51.7)	70 (63.6)	23 (67.6)	
≥37 (N, %)	23 (38.3)	16 (14.5) [a]	2 (5.9) [a]	
**Premature (N, %)**	37 (61.7)	94 (85.5) [a]	32 (94.1) [a]	**<0.001**
**Low birth weight (N, %)**	15 (25.0)	46 (41.8)	19 (55.9) [a]	**0.009**
**Asphyxia (N, %)**	7 (11.7)	28 (25.5)	7 (20.6)	0.105
**NICU administration (N, %)**	21 (35.0)	85 (77.3) [a]	28 (82.4) [a]	**<0.001**
**Length of NICU [Media, (P25, P75)]**	0.00 [0.00, 8.00]	8.00 [6.00, 15.75] [a]	14.00 [6.25, 17.00] [a]	**<0.001**
**Apgar -1 min [Media, (P25, P75)]**	10.00 [9.00, 10.00]	8.00 [6.00, 10.00] [a]	7.50 [6.00, 10.00] [a]	**0.001**
**Apgar-5 mins [Media, (P25, P75)]**	10.00 [10.00, 10.00]	10.00 [9.00, 10.00] [a]	10.00 [9.00, 10.00]	**0.008**
**Apgar-10 mins [Media, (P25, P75)]**	10.00 [10.00, 10.00]	10.00 [10.00, 10.00]	10.00 [10.00, 10.00]	0.286

[a] *P* <0.05 vs 0CSh; [b] *P* <0.05 vs 1CSh.

Table S7. Association between maternal cesarean section history and adverse neonatal outcomes (adjusted for gestational age)

	0CSh<br>N=60	1CSh<br>N=110		≥2CSh<br>N=34	
		OR (95%CI)	aOR (95%CI)	OR (95%CI)	aOR (95%CI)
**Preterm delivery**	ref	3.65 (1.74, 7.68)	2.6 (1.13, 5.96)	9.95 (2.17, 45.49)	5.96 (1.24, 28.75)
**Low birth weight**	ref	2.16 (1.07, 4.33)	0.95 (0.37, 2.41)	3.80 (1.55, 9.29)	1.38 (0.43, 4.39)
**Asphyxia**	ref	2.59 (1.05, 6.34)	1.35 (0.49, 3.72)	1.96 (0.62, 6.17)	0.77 (0.21, 2.83)
**NICU administration**	ref	6.31 (3.16,12.63)	**3.42 (1.39, 8.42)**	8.67 (3.10, 24.25)	2.89 (0.82, 10.18)

OR, odds ratio; CI, confidence interval; NICU, neonatal intensive care unit adjusted for maternal age ≥35 years, placenta previa, current ART, and gestational age at delivery.

STROBE Statement—Checklist of items that should be included in reports of ***cohort studies***

	Item No	Recommendation	Page No
**Title and abstract**	1	(*a*) Indicate the study's design with a commonly used term in the title or the abstract<br>(*b*) Provide in the abstract an informative and balanced summary of what was done and what was found	1
**Introduction**			
Background/rationale	2	Explain the scientific background and rationale for the investigation being reported	2
Objectives	3	State specific objectives, including any prespecified hypotheses	2
**Methods**			
Study design	4	Present key elements of study design early in the paper	3
Setting	5	Describe the setting, locations, and relevant dates, including periods of recruitment, exposure, follow-up, and data collection	3
Participants	6	(*a*) Give the eligibility criteria, and the sources and methods of selection of participants. Describe methods of follow-up<br>(*b*) For matched studies, give matching criteria and number of exposed and unexposed	3
Variables	7	Clearly define all outcomes, exposures, predictors, potential confounders, and effect modifiers. Give diagnostic criteria, if applicable	3
Data sources/ measurement	8*	For each variable of interest, give sources of data and details of methods of assessment (measurement). Describe comparability of assessment methods if there is more than one group	4
Bias	9	Describe any efforts to address potential sources of bias	4
Study size	10	Explain how the study size was arrived at	3
Quantitative variables	11	Explain how quantitative variables were handled in the analyses. If applicable, describe which groupings were chosen and why	4
Statistical methods	12	(*a*) Describe all statistical methods, including those used to control for confounding<br>(*b*) Describe any methods used to examine subgroups and interactions<br>(*c*) Explain how missing data were addressed<br>(*d*) If applicable, explain how loss to follow-up was addressed<br>(*e*) Describe any sensitivity analyses	4-5
**Results**			
Participants	13*	(a) Report numbers of individuals at each stage of study—eg numbers potentially eligible, examined for eligibility, confirmed eligible, included in the study, completing follow-up, and analysed<br>(b) Give reasons for non-participation at each stage<br>(c) Consider use of a flow diagram	6
Descriptive data	14*	(a) Give characteristics of study participants (eg demographic, clinical, social) and information on exposures and potential confounders<br>(b) Indicate number of participants with missing data for each variable of interest<br>(c) Summarise follow-up time (eg, average and total amount)	6
Outcome data	15*	Report numbers of outcome events or summary measures over time	7

Main results	16	(*a*) Give unadjusted estimates and, if applicable, confounder-adjusted estimates and their precision (eg, 95% confidence interval). Make clear which confounders were adjusted for and why they were included<br>(*b*) Report category boundaries when continuous variables were categorized<br>(*c*) If relevant, consider translating estimates of relative risk into absolute risk for a meaningful time period	6-8
Other analyses	17	Report other analyses done—eg analyses of subgroups and interactions, and sensitivity analyses	Supplementary
**Discussion**			
Key results	18	Summarise key results with reference to study objectives	8
Limitations	19	Discuss limitations of the study, taking into account sources of potential bias or imprecision. Discuss both direction and magnitude of any potential bias	9-10
Interpretation	20	Give a cautious overall interpretation of results considering objectives, limitations, multiplicity of analyses, results from similar studies, and other relevant evidence	8
Generalisability	21	Discuss the generalisability (external validity) of the study results	8-9
**Other information**			
Funding	22	Give the source of funding and the role of the funders for the present study and, if applicable, for the original study on which the present article is based	11

*Give information separately for exposed and unexposed groups.

Note: An Explanation and Elaboration article discusses each checklist item and gives methodological background and published examples of transparent reporting. The STROBE checklist is best used in conjunction with this article (freely available on the Web sites of PLoS Medicine at http://www.plosmedicine.org/, Annals of Internal Medicine at http://www.annals.org/, and Epidemiology at http://www.epidem.com/). Information on the STROBE Initiative is available at http://www.strobe-statement.org.
